# Association Between Antibiotic Treatment of *Chlamydia pneumoniae* and Reduced Risk of Alzheimer Dementia: A Nationwide Cohort Study in Taiwan

**DOI:** 10.3389/fnagi.2021.701899

**Published:** 2021-08-18

**Authors:** Hsun Ou, Wu-Chien Chien, Chi-Hsiang Chung, Hsin-An Chang, Yu-Chen Kao, Pei-Chuan Wu, Nian-Sheng Tzeng

**Affiliations:** ^1^Department of Psychiatry, Tri-Service General Hospital, School of Medicine, National Defense Medical Center, Taipei, Taiwan; ^2^Department of Medical Research, Tri-Service General Hospital, National Defense Medical Center, Taipei, Taiwan; ^3^School of Public Health, National Defense Medical Center, Taipei, Taiwan; ^4^Graduate Institute of Life Sciences, National Defense Medical Center, Taipei, Taiwan; ^5^Taiwanese Injury Prevention and Safety Promotion Association, Taipei, Taiwan; ^6^Student Counseling Center, National Defense Medical Center, Taipei, Taiwan; ^7^Department of Psychiatry, Tri-Service General Hospital, Song-Shan Branch, National Defense Medical Center, Taipei, Taiwan; ^8^Department of Psychiatry, Far Eastern Memorial Hospital, New Taipei City, Taiwan

**Keywords:** *Chlamydia pneumoniae*, Alzheimer dementia, National Health Insurance Research Database, macrolide, fluoroquinolone, nationwide cohort study

## Abstract

**Background:***Chlamydia pneumoniae* (CPn) is a common community-acquired pneumonia. In the literature, CPn infection is demonstrated to exhibit an association with Alzheimer dementia (AD). We executed the present nationwide, population-based research with the goal of probing the association of CPn infection and antibiotic therapy with AD risk.

**Methods:** We conducted a cohort study using a database extracted from Taiwan's National Health Insurance Research Database (NHIRD). All medical conditions for each enrolled individuals were categorized using the International Classification of Diseases, ninth Revision classifications. Hazard ratios (HRs) and 95% confidence intervals (CIs) for associations between CPn pneumonia-associated hospitalizations and AD were estimated using Fine and Gray's survival analysis and adjusted for comorbidities. The effects of the antibiotics on the HRs for AD in the patients with CPn pneumonia-associated hospitalization were also analyzed.

**Results:** Our analyses included 6,628 individuals, including 1,657 CPn-infected patients, as well as 4,971 controls matched by age, index date, and sex (1:3). In this study, patients hospitalized for CPn pneumonia exhibited a significantly higher AD risk (adjusted HR = 1.599, 95% CI = 1.284–1.971, *p* < 0.001). We also noted an association of macrolide use (≥15 days) and fluoroquinolone use (≥15 days) with decreased AD risk.

**Conclusions:** We determined CPn pneumonia to be associated with a relatively high AD risk. The result in this study confirmed the findings from previous literatures, by using a large, nationwide, population-based database. Appropriate macrolide and fluoroquinolone treatment may attenuate this risk.

## Introduction

Dementia is one of the major global health problems. In Taiwan, the prevalence is 4–8% for a population aged≥65 in community studies (Sun et al., 2014), and it is therefore considered a heavy burden for the family, the caregivers, and society of these patients (Tzeng et al., 2015). Of the assorted varieties of dementia, Alzheimer dementia (AD) constitutes the most common, with an etiology that remains unclear; however, it is thought that the combined presence of amyloid and tau proteins and some genetic factors contribute to the pathogenesis of AD.

Despite decades of research, much of the heritability of AD remains unexplained. Genes may play a role in some cases of AD. Most of the previous studies have indicated that the development of AD is not attributable to one or even a few mutations or gene polymorphisms. Instead, the disease genesis is considered multifactorial and may result in unknown environmental and host genetic factors (Balin et al., 2018).

Recently, research focused on the relationship between neuroinflammation and AD. These studies indicated that inflammation would lead to neuronal damage and death in the brain. One study also demonstrated that neuroinflammation altered the expression and activity of amyloid precursor protein and promote amyloid formation (Al-Atrache et al., 2019). Additionally, microbial infections are believed to play a role in the development of neuroinflammation and increased the risk of AD (Boelen et al., 2007; Bloom, 2014; Al-Atrache et al., 2019).

Among various microbial infections, researchers have proposed *Chlamydia pneumoniae* (CPn) may be implicated in AD pathology and certain antibiotics were determined to exhibit some degree of effectiveness in treating moderate AD (Loeb et al., 2004); however, the results of human and animal studies in past decades were inconsistent (Maheshwari and Eslick, 2015; Woods et al., 2020). Because nationwide population-based studies have yet to be executed to corroborate the CPn infection–AD association. Accordingly, in our attempt to bridge this gap, we executed our current study by employing information gleaned from the National Health Insurance Research Database (NHIRD) of Taiwan. The aim of this study is to determine the association between CPn pneumonia, antibiotics therapy, and the risk of AD.

## Methods

### Data Sources

Data for our nationwide, population-based, cohort study were obtained from the inpatient care records and registration files from the NHIRD. The National Health Insurance (NHI) program was implemented in 1995 and provides healthcare coverage to 99% of the population (being more than 23 million people). The NHI Administration randomly reviews the records of ambulatory care visits and in-patient claims periodically so as to verify the accuracy of the diagnoses (National Health Insurance Administration National Health Insurance Regulations). The details of the program have been documented in previous studies (Chang et al., 2018; Chao et al., 2018). It should be noted that some studies have demonstrated the accuracy and validity of several diagnoses in the NHIRD, including diabetes mellitus (DM), cancer, myocardial infarction, and the central nervous system diseases, such as Tourette syndrome, and stroke.

### Study Population

This study involved a cohort design. Using the NHIRD, we selected adult patients aged >50 years who had been diagnosed with AD (331.0) based on the International Classification of Diseases, 9th Revision, Clinical Modification (ICD-9-CM) codes between 2000 and 2015, and confirmed these patients' diagnoses by linking the records of at least three visits for AD in consecutive years, or at least one hospitalization for AD, during the study period. All diagnoses of AD were made by board-certified psychiatrists or neurologist in Taiwan. The date of the AD diagnosis was at least 1 year after the CPn pneumonia diagnosis. We identified patients with CPn pneumonia using the ICD-9-CM code 483.1. We confirmed these patients' diagnoses by linking the records of at least one hospitalization for CPn pneumonia, in the study period, and the reason why we decided on the enrolled exposures of CPn as pneumonia due to CPn, was because one previous population-based study had confirmed the accuracy of the diagnosis of pneumonia from the NHIRD as 98.3% (Su et al., 2014). For each patient with Cpn pneumonia included in our study, three controls were selected via 1:3 matching by age, sex, and the number of medical follow-ups (N = 4971) in the NHIRD. All insurance claims were scrutinized by medical reimbursement specialists, and peer reviews were undertaken according to the standard and clinical diagnostic criteria.

### Covariates

The covariates included sex, age groups (50–64, ≥65 years), marital status, education (<12 years,≥12 years), seasons, geographical area of residence (north, center, south, and east of Taiwan), urbanization level of residence (levels 1–4), and monthly income (in New Taiwan Dollars [NT$]; <18,000, 18,000–34,999, ≥35,000). The urbanization level of residence was defined according to the population and various indicators of the level of development. Level 1 was defined as a population of >1,250,000, and a specific designation as political, economic, cultural, and metropolitan development. Level 2 was defined as a population between 500,000 and 1,249,999, and as playing a key role in the politics, economy, and culture. Urbanization levels 3 and 4 were defined as a population between 149,999 and 499,999, and <149,999, respectively (Chang et al., 2014).

Data on the usage of macrolides and fluoroquinolones antibiotics were acquired from the Longitudinal Health Insurance Database (LHID), a sub-database of the NHIRD. The data of the defined daily dosage (DDD) were obtained from the WHO Collaborating Centre for Drug Statistics Methodology (https://www.whocc.no/), and the duration of the usage of antibiotics was calculated by dividing the cumulative dosages by the DDD of the antibiotics.

### Comorbidity

The comorbidities, including DM (ICD-9-CM 250), hypertension (ICD-9-CM 401.1, 401.9, 402.10, 402.90, 404.10, 404.90, 405.1, and 405.9), hyperlipidemia (ICD-9-CM 272), coronary artery disease (CAD, ICD-9-CM code 410–414), obesity (ICD-9-CM 278), all cancers (ICD-9-CM 140–208), and other chlamydia infections (ICD-9-CM codes: 077.98, ICD-9-CM 078.88, 079.88, 079.98, 099.41, and 099.5) and other pneumonia (ICD-9-CM codes: 480–486, except 483.1). These comorbidities were included with the references from previous studies using health databases (Wright et al., 2015; Gottesman et al., 2017).

The Charlson comorbidity index (CCI) was employed to execute the assessment of the aforementioned comorbidities. In the CCI, *ICD-9-CM* codes are used as the basis for the establishment of categories for comorbidities, and each of the established categories is scored (van den Berg et al., 2013, 2014; Wong et al., 2014); to derive a single comorbidity score, all CCI scores are combined, with 0 indicating no comorbidities and higher scores (1, 2, 3, ≥4) indicating higher comorbidity burdens (Needham et al., 2005).

### Definitions of Patients With CPn Pneumonia

Only patients diagnosed with CPn pneumonia of more than 1 year prior to the index date were considered. CPn pneumonia was identified from the NHIRD by using the corresponding ICD-9 code (ICD-9-CM code 483.1). All the covariates as aforementioned were included.

### Statistical Analysis

Categorical variables, which were presented as percentages, were compared using the χ^2^ tests and the Fisher's exact test. Continuous variables, which were presented as the mean and SD, were compared using the Student's *t*-tests. The primary goal of this study was to determine as to whether a patient's clinical characteristics, such as CPn pneumonia, were associated with AD. Associations between those outcomes and clinical characteristics were investigated using the Fine and Gray's survival analysis in a generalized estimating equation (GEE) model. The regression results are presented as adjusted HRs with corresponding 95% CIs. The threshold for statistical significance was *p* < 0.05. All data analyses were conducted using the SPSS V.22 (SPSS).

## Results

### Enrolled Samples

Supplementary Figure 1 is a flowchart of the patient enrollment procedure. From the NHIRD, we identified 1,657 patients who received a CPn pneumonia diagnosis during our defined study period (2000–2015); these patients were matched 1:3 with patients without CPn (N = 4,971) according to age, sex, number of visits to medical facilities, and comorbidities.

### Sample Characteristics

In total, 1,657 patients were diagnosed with Cpn pneumonia during the study period. A total of 182 enrollee were identified with AD during the follow-up period.

Table 1 shows the sex, age, marital status and comorbidities of the patients with Cpn pneumonia. When compared with controls, the patients with Cpn pneumonia tended to have higher rate of hypertension, hyperlipidemia, other pneumonia, anxiety, sleep disorder and CCI scores.

**Table 1:** Characteristics of study at the baseline.

***Chlamydia pneumoniae***	**With**		**Without**		***p***
**Variables**	**n**	**%**	**n**	**%**	
**Total**	1,657	25.00	4,971	75.00	
**Gender**					0.999
Male	1,002	60.47	3,006	60.47	
Female	655	39.53	1,965	39.53	
**Age (years)**	65.53 ± 10.13		65.71 ± 9.17		0.495
**Age groups (years)**					0.999
50-64	809	48.82	2,427	48.82	
≥65	848	51.18	2,544	51.18	
**Marital status**					0.681
Without	809	48.82	2,456	49.41	
With	848	51.18	2,515	50.59	
**Education (years)**					0.977
<12	892	53.83	2,674	53.79	
≥12	765	46.17	2,297	46.21	
**Insured premium (NT$)**					<0.001
<18,000	1,607	96.98	4,926	99.09	
18,000-34,999	41	2.47	43	0.87	
≥35,000	9	0.54	2	0.04	
**Diabetes mellitus**	275	16.60	838	16.86	0.820
**Hypertension**	416	25.11	988	19.88	<0.001
**Hyperlipidemia**	77	4.65	171	3.44	0.030
**Coronary artery disease**	178	10.74	587	11.81	0.249
**Obesity**	0	0.00	2	0.04	0.414
**Cancer**	122	7.36	400	8.05	0.400
**Pneumonia**	252	15.21	541	10.88	<0.001
**Depression**	11	0.66	24	0.48	0.433
**Bipolar**	3	0.18	9	0.18	0.999
**Anxiety**	465	28.06	1,230	24.74	0.008
**Alcohol use disorder**	9	0.54	15	0.30	0.160
**Substance use disorder**	1	0.06	2	0.04	0.739
**Sleep disorder**	20	1.21	19	0.38	0.001
***Other Chlamydiae*** **infections or diseases**	1	0.06	0	0.00	0.250
**CCI_R**					<0.001
0	1,067	64.39	3,199	64.35	
1	440	26.55	1,126	22.65	
2	105	6.34	414	8.33	
3	28	1.69	153	3.08	
≥4	17	1.03	79	1.59	
**Season**					0.172
Spring (Mar–May)	453	27.34	1,287	25.89	
Summer (Jun–Aug)	395	23.84	1,127	22.67	
Autumn (Sep–Nov)	363	21.91	1,082	21.77	
Winter (Dec–Feb)	446	26.92	1,475	29.67	
**Location**					<0.001
Northern Taiwan	742	44.78	1,850	37.22	
Middle Taiwan	458	27.64	1,413	28.42	
Southern Taiwan	287	17.32	1,325	26.65	
Eastern Taiwan	167	10.08	352	7.08	
Outlets islands	3	0.18	31	0.62	
**Urbanization level**					<0.001
1 (The highest)	570	34.40	1,549	31.16	
2	844	50.94	2,188	44.02	
3	84	5.07	346	6.96	
4 (The lowest)	159	9.60	888	17.86	
**Level of care**					<0.001
Hospital center	591	35.67	1,542	31.02	
Regional hospital	749	45.20	1,573	31.64	
Local hospital	317	19.13	1,856	37.34	

*CCI_R: Charlson Comorbidity Index, dementia removed; P: Chi-square/Fisher exact test on category variables and t-test on continue variables*.

Patients with CPn pneumonia also tended to be living in northern and middle Taiwan and residing more in the regions of urbanization levels 1 and 2. There were no differences in the distribution of sex, age, marital status, and education between these two groups.

### Hazard Ratios Analysis of AD in the Patients With CPn Pneumonia

In our applied Fine & Gray's competing risk model, compared with the control group, we determined patients with CPn pneumonia to have a higher risk of AD (adjusted hazard ratio [HR] = 1.599, 95% CI = 1.284–1.971, *p* < 0.001; Table 2) after we adjusted for urbanization level/geographic region, sex, marital status, education, comorbidities (including CCI scores), age, antibiotic use, and insurance premium.

**Table 2:** Factors for Alzheimer dementia by using the analysis of Fine and Gray's competing risk model.

	**Competing risk in the model**							
**Variables**	**Crude HR**	**95% CI**	**95% CI**	***P***	**Adjusted HR**	**95% CI**	**95% CI**	***P***
*Chlamydia pneumonia (reference: without)*	1.676	1.425	1.902	<0.001	1.599	1.284	1.971	<0.001
Male (*reference: female*)	1.182	1.007	1.387	0.041	1.164	0.989	1.369	0.067
Hyperlipidemia (*reference: without*)	1.497	1.298	1.830	<0.001	1.427	1.252	1.724	0.002
Coronary artery disease (*reference: without*)	1.492	1.396	1.657	<0.001	1.478	1.357	1.640	<0.001
Hypertension (*reference: without*)	0.843	0.713	1.007	0.064	1.060	0.723	1.222	0.087
Cancer (*reference: without*)	0.376	0.265	0.535	<0.001	0.400	0.280	0.572	<0.001
Depression (*reference: without*)	2.066	1.106	3.860	0.023	1.551	0.825	2.917	0.173
Anxiety (*reference: without*)	1.627	1.333	1.901	<0.001	1.638	1.394	1.926	<0.001
Alcohol use disorder (*reference: without*)	5.351	2.001	14.311	0.001	5.778	2.138	15.637	0.001
Sleep disorder (*reference: without*)	2.090	1.180	3.701	0.011	2.011	1.129	3.584	0.018
CCI_R 1 (*reference: CCI_R: 0*)	1.546	1.303	1.835	<0.001	1.353	1.134	1.615	0.001
CCI_R 2 (*reference: CCI_R: 0*)	1.348	1.036	1.745	0.026	1.081	0.826	1.415	0.599
CCI_R 3 (*reference: CCI_R: 0*)	1.759	1.201	2.576	0.004	1.436	0.976	2.113	0.066
Autumn (*reference: Spring*)	0.744	0.593	0.933	0.011	0.704	0.560	0.885	0.003
Medical center (*reference: local hospital*)	1.689	1.570	1.832	<0.001	1.692	1.569	1.842	<0.001
Regional hospital (*reference: local hospital*)	1.620	1.503	1.764	<0.001	1.645	1.510	1.815	<0.001

*HR, hazard ratio; CI, confidence interval; Adjusted HR, adjusted variables listed in the Table 1; CCI_R, Charlson Comorbidity Index, dementia removed; P: Chi-square/Fisher exact test on category variables and t-test on continue variable*.

In addition, we observed patients with hyperlipidemia, CAD, anxiety, sleep disorder, and alcohol use disorder to be at a relatively high risk of AD. Furthermore, we noted those receiving care from a medical center or regional hospital to exhibit a relatively high risk of AD. Conversely, patients who sought medical care in Autumn and with cancer revealed a reduced risk of AD. Although hypertension exhibited a significant difference in the patients with CPn pneumonia group at baseline (see in Table 1), it did not significantly affect developing AD by the logistic regression model (*p* = 0.087; Table 2).

### Antibiotics and the Risk of AD in Patients With CPn Pneumonia

Table 3 shows the usage of antibiotics in the risk of AD in patients with CPn pneumonia, and that the usage of macrolides (≥15 days) and fluoroquinolones (≥15 days), were associated with a decreased risk of dementia.

**Table 3:** Factors for Alzheimer dementia among different usages of antibiotics by using Fine and Gray's competing risk model.

**Medications**		**Adjusted HR**	**95% CI**	**95% CI**	***P***
Macrolides	Without *Chlamydia pneumoniae*	Reference			
	With *Chlamydia pneumoniae*				
	Without Macrolides	2.177	1.865	2.468	<0.001
	with Macrolides 1–7 days	1.483	1.069	1.825	0.003
	with Macrolides 8–14 days	1.592	1.276	1.899	<0.001
	with Macrolides≥15 days	1.198	0.929	1.487	0.304
Fluoroquinolones	Without *Chlamydia pneumoniae*	Reference			
	With *Chlamydia pneumoniae*				
	Without Fluoroquinolones	1.996	1.395	2.896	<0.001
	With Fluoroquinolones 1-7 days	1.506	1.283	1.883	<0.001
	With Fluoroquinolones 8-14 days	1.385	1.106	1.796	0.001
	With Fluoroquinolones≥15 days	1.302	0.973	1.656	0.391

*PYs, Person-years; Adjusted HR, Adjusted variables listed in the table; CI, confidence interval; P, Chi-square/Fisher exact test on category variables and t-test on continue variable*.

### Kaplan–Meier Curves for the Cumulative Incidence of AD in Patients With CPn Pneumonia

**Figure 1:**
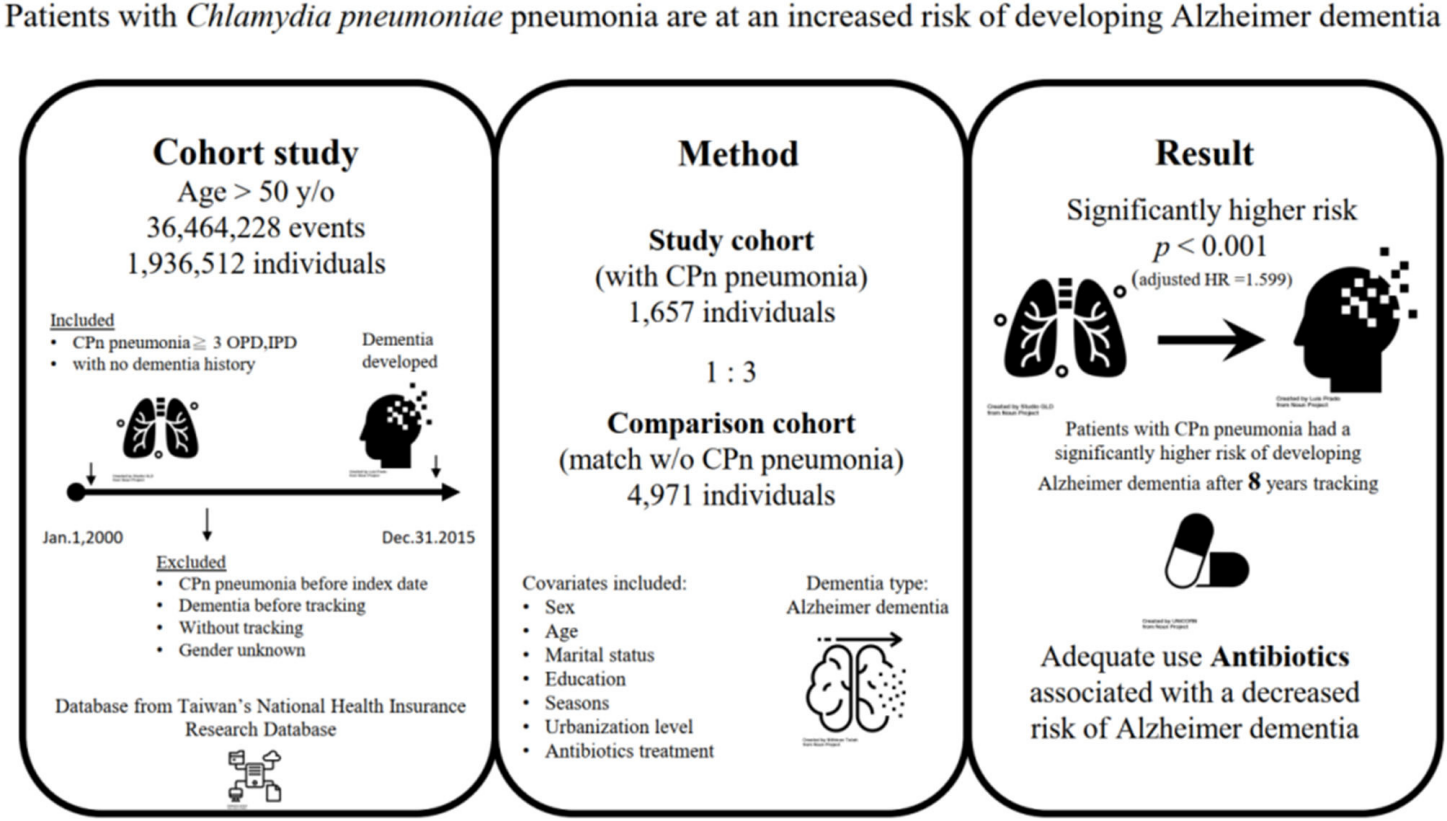
The graphic abstract of study design and results from National Health Insurance Research Database in Taiwan. CPn, *Chlamydia pneumoniae*; OPD, outpatient clinic; IPD, Inpatient departments; HR, Hazard Ratio. All icons are from the Noun Project.

The cumulative incidences of AD were 931.01 per 10^5^ person-years and 764.22 per 10^5^ person-years, in the study cohort and comparison cohort group, respectively (Supplementary Table 1). The difference between the two groups was significant (log-rank test, *p* < 0.001; Figure 1). After 8 years of tracking, the cumulative incidence of AD in patients with CPn pneumonia and comparison group was significant (log-rank test, *p* < 0.037; Figure 1).

## Discussion

### CPn Pneumonia–AD Risk Association

By employing a nationwide population database, our executed study is the first to demonstrate an association between CPn pneumonia and AD development in Taiwan. Patients with CPn pneumonia exhibited a nearly 1.6-fold increased risk of AD (overall adjusted HR = 1.599; 95% CI = 1.284–1.971, *p* < 0.001). Additionally, hyperlipidemia, CAD, anxiety, sleep disorder, and alcohol use disorder exhibited associations with a relatively high risk of AD. Patients who visited medical centers or regional hospitals to seek medical care were also noted to be at relatively high risk. Although the percentage of hypertension in CPn exposed cohort was higher than that in the unexposed cohort (*p* < 0.001), in Fine & Gray's survival analysis, there were no significant differences for hypertension between the two cohorts (*p* = 0.087). However, patients who sought medical care in Autumn and with cancer revealed a reduced risk of AD. Further studies are needed to clarify why patients with these comorbidities were associated with a decreased risk. Moreover, our study also indicated that patients who were treated with macrolides (≥15 days) and fluoroquinolones (≥15 days) could attenuate the risk of AD.

### Comparison of This Study to Previous Literatures

Some previous studies have found that CPn was associated with AD (Balin et al., 1998; Gerard et al., 2006; Paradowski et al., 2007), but other studies had failed to detect such an association (Taylor et al., 2002; Wozniak et al., 2003; Yamamoto et al., 2005; Hammond et al., 2010). In one meta-analysis—the procedures of which entailed pooling cases and controls in previous studies—chlamydial infection was noted to exhibit an association with a 5-fold rise in the AD occurrence (OR: 5.66; 95% CI = 1.83–17.51, *p* < 0.001) (Maheshwari and Eslick, 2015). In the present study, because the pneumonia diagnosis as recorded in the NHIRD was determined to have 98.3% accuracy (Su et al., 2014), we restricted patients with CPn infection to those with CPn pneumonia to bolster the credibility of the association between AD and CPn.

### How CPn Pneumonia Might Increase the Risk of AD

The first formal characterization of AD was made in 1907; nevertheless, its corresponding etiology remains poorly understood. AD is commonly regarded as a neurodegenerative disease and is ascribed to neuronal damage and death. Neuropathology in AD is characterized by neuropil threads, neurofibrillary tangles, neurotic senile plaques, and typically amyloid deposits around the cerebral vasculature. Neuroinflammatory processes, oxidative stress, and vascular factors are the three main contributors to AD pathogenesis (Ashraf et al., 2019).

**Figure 2:**
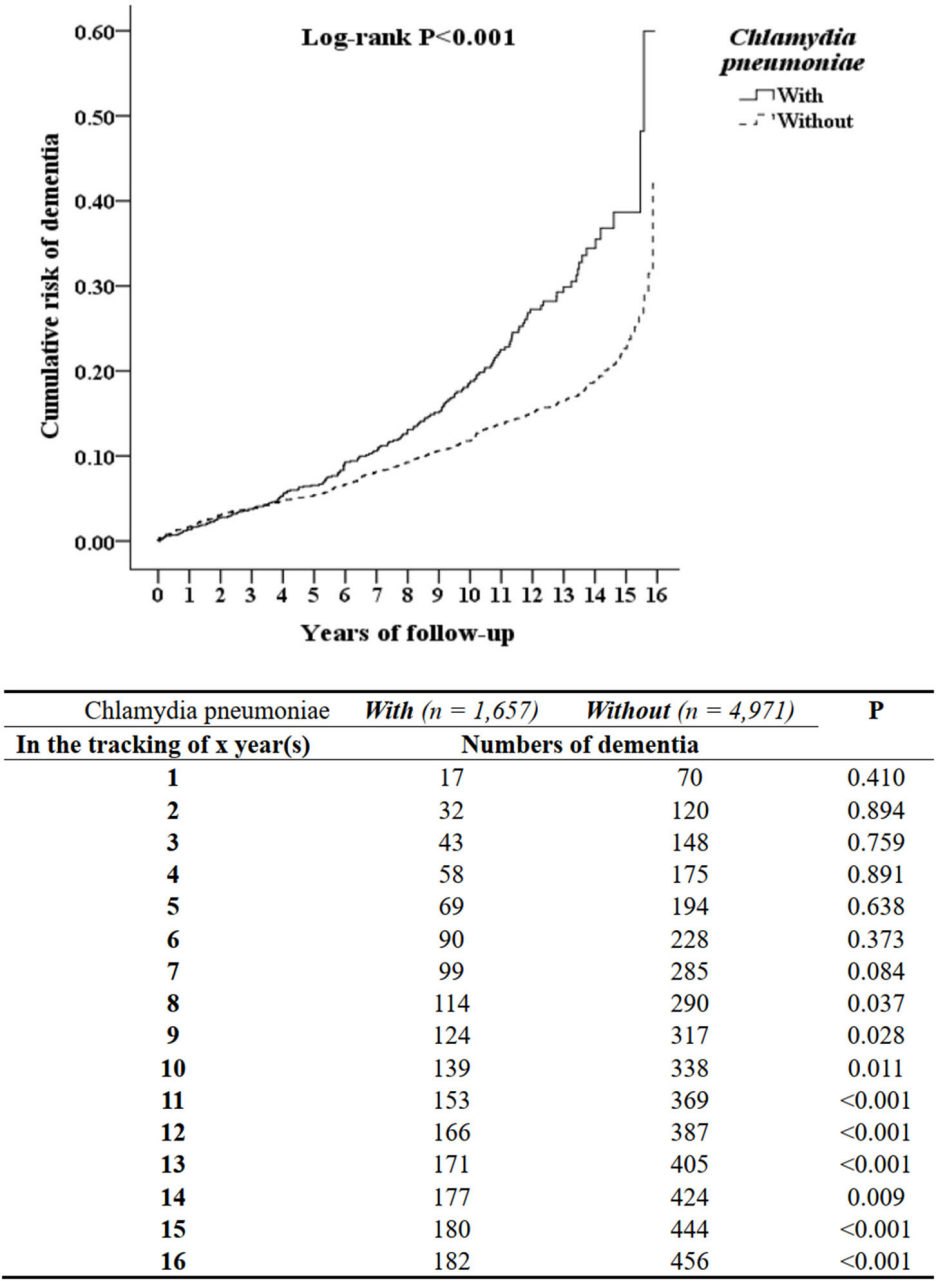
Kaplan-Meier for cumulative risk of dementia aged 50 and over stratified by *Chlamydia pneumoniae* with log-rank test.

Many studies have confirmed the relationship between neuroinflammation and AD, despite the mechanistic links requiring further investigation. Herpes simplex type 1, human herpesvirus 6, *Borrelia burgdorferi*, and *Helicobacter pylori* are among the myriad of pathogens that may be involved in AD (Mawanda and Wallace, 2013). Neuroinflammation in the brain may contribute to AD pathogenesis. Among various pathogens, researchers have proposed CPn may cause chronic neuroinflammation and be implicated in AD. CPn infection of endothelial cells—infection at the vascular level—could engender increased monocyte migration and advance inflammation in the CNS (MacIntyre et al., 2003). Moreover, in THP1 human monocytes, CPn infection stimulates an innate immune response, and thus, such infection may have a role in inflammation initiation in cases of sporadic/late-onset Alzheimer disease (Lim et al., 2014). Some animal studies have determined that CPn is related to beta-amyloid (Aβ) 1–42 immunoreactive deposits in rodent brain tissues (Little et al., 2004; Boelen et al., 2007). A recent *in vitro* research claimed that CPn infection also altered the expression and activity of amyloid precursor protein and promoted amyloid formation (Al-Atrache et al., 2019). These findings provided evidence for a link between CPn and AD pathology.

According to the antimicrobial protection hypothesis (Moir et al., 2018), Aβ oligomerization is not inherently pathological; Aβ deposition represents an early innate immune reaction to an immunochallenge, whether genuine or mistakenly perceived. Aβ fibrillization inactivates neuroinflammatory pathways to defend against infection and clear the deposition of Aβ or pathogens. In cases of AD, sustained inflammation and neurodegeneration result from chronic activation of the pathway. It could be explained the cumulative incidence of AD in patients with CPn pneumonia exhibited significant after 8 years of tracking in this study.

In addition, the apolipoprotein E4 (APOE) genotype may be involved in the CPn–AD association (Wehr et al., 2006). Scholars have determined the APOE gene to be involved in cerebral Aβ clearance, with CPn influencing neuronal damage repair ability (Masters et al., 2015; Woods et al., 2020). Hence, another possible mechanism underlying CPn toxicity in AD may be the interaction of the APOE genotype with CPn.

As revealed in the literature, antibiotics against CPn were noted to decrease the deposition of amyloids, oxidative stress, and inflammation in AD models (Yulug et al., 2018; Balducci and Forloni, 2019); these antibiotics possess the capacity to traverse the blood–brain barrier. In this study, we noted macrolide use (≥15 days) and fluoroquinolone use (≥15 days) to exhibit an association with a decrease in dementia risk. Compared with macrolides, fluoroquinolones are much more lipophilic and smaller and are hence more efficacious when administered in CNS. Macrolides cannot cross the blood–brain barrier, but they are usually the first-line therapy for CPn infection; no evidence has suggested macrolides are neurotoxic or affect AD risk, and it is presumed such drugs (i.e., azithromycin, clarithromycin) do not exacerbate AD (Woods et al., 2020). The role of antibiotics against CPn and the risk of AD requires further investigation.

Pneumonia caused by CPn is typically mild, and most patients recover without complications. Our study indicated that the appropriate use of antibiotics with macrolides and fluoroquinolones (both duration≥15 days) could decrease the risk of developing AD. In one clinical practice guideline, continue antibiotic therapy is strongly recommended in patients with CPn infection until they achieve clinical stability including resolution of vital sign abnormalities, appetite, and normal mentation (Metlay et al., 2019).

CPn is a type of community-acquired pneumonia and is transmitted person-to-person through inhalation of respiratory droplets or contact with droplets on surfaces followed by contact (i.e., touching) with the mouth or nose. CPn reinfection among older adults is common, and outbreaks have been reported in settings of close contact and crowding, such as nursing homes, schools, prisons, and military barracks (Burillo and Bouza, 2010). This transmissibility may explain why we noted an association between a high AD risk undergoing medical care at regional hospitals or medical centers.

### Limitations

The NHIRD recorded inpatient care, ambulatory care, dental care, and prescription drugs availed by the insured and their date of birth. However, pursuant to the Personal Information Protection Act, individual identifiers are encrypted before releasing for research.

Therefore, information such as weakness severity, laboratory parameters, neurological symptom severity, additional examination findings (e.g., electrophysiological testing), or rehabilitation availability could not be assessed in our executed study due to the lack of such data in the NHIRD.

Besides, we could not include data on psychosocial, environmental, and genetic factors in our analyses due to the same reason. However, despite these limitations, our derived data are highly likely to be valid and representative due to the NHIRD containing data covering all hospitals within Taiwan and over 99% of the population for the relevant 15-year period.

## Conclusions

This study determined CPn pneumonia to be associated with an estimated 1.6-fold increased risk of AD, which should alert physicians to be attentive to the risk of AD following CPn pneumonia, especially after 8 years of tracking. We noted the AD risk to be reduced among CPn pneumonia patients when administered appropriate antibiotics. We recommend the execution of additional studies based on extensive or national data sets to corroborate the present findings and elucidate the corresponding underlying mechanisms.

## Data Availability Statement

The data on the study population that were obtained from the NHIRD (http://nhird.nhri.org.tw/en/index.html) are maintained in the NHIRD (http://nhird.nhri.org.tw/). The NHRI is a nonprofit foundation established by the government. Only citizens of Taiwan who fulfill the requirements of conducting research projects are eligible to apply for access to the NHIRD. The use of the NHIRD is limited to research purposes only. Applicants must follow the Computer-Processed Personal Data Protection Law (http://www.winklerpartners.com/?p=987) and the related regulations of the National Health Insurance Administration and NHRI, and an agreement must be signed by the applicant and their supervisor upon application submission. All applications are reviewed for approval of data release.

## Ethics Statement

The studies involving human participants were reviewed and approved by The Institutional Review Board of the Tri-Service General Hospital (IRB No. 2-107-05-026). Written informed consent for participation was not required for this study in accordance with the national legislation and the institutional requirements.

## Author Contributions

The contributions of the authors are listed as the following: HO and N-ST: study concept and design. W-CC, C-HC, and N-ST: acquisition of data. W-CC, C-HC, H-AC, Y-CK, P-CW, and N-ST: analysis and interpretation of data. HO: drafting of the manuscript. N-ST: critical revision of the manuscript for important intellectual content. All authors contributed to the article and approved the submitted version.

## Conflict of Interest

The authors declare that the research was conducted in the absence of any commercial or financial relationships that could be construed as a potential conflict of interest.

## Publisher's Note

All claims expressed in this article are solely those of the authors and do not necessarily represent those of their affiliated organizations, or those of the publisher, the editors and the reviewers. Any product that may be evaluated in this article, or claim that may be made by its manufacturer, is not guaranteed or endorsed by the publisher.

## Abbreviations

AD: Alzheimer dementia
APOE: apolipoprotein E4
CI: confidence interval
CCI: Charlson comorbidity index
CAD: coronary artery disease
CNS: central nervous system
CPn: *Chlamydia pneumoniae*
DM: diabetes mellitus
DDD: defined daily dosage
LHID: longitudinal Health Insurance Database
NHIRD: National Health Insurance Research Database
NHI: National Health Insurance
HR: hazard ratio.
