# Supplementary figures and images for: Association Between Antibiotic Treatment of Chlamydia pneumoniae and Reduced Risk of Alzheimer Dementia: A Nationwide Cohort Study in Taiwan
Source: Front Aging Neurosci. 2021 Aug 18;13:701899. doi: 10.3389/fnagi.2021.701899 (PMC8416516; doi:10.3389/fnagi.2021.701899)

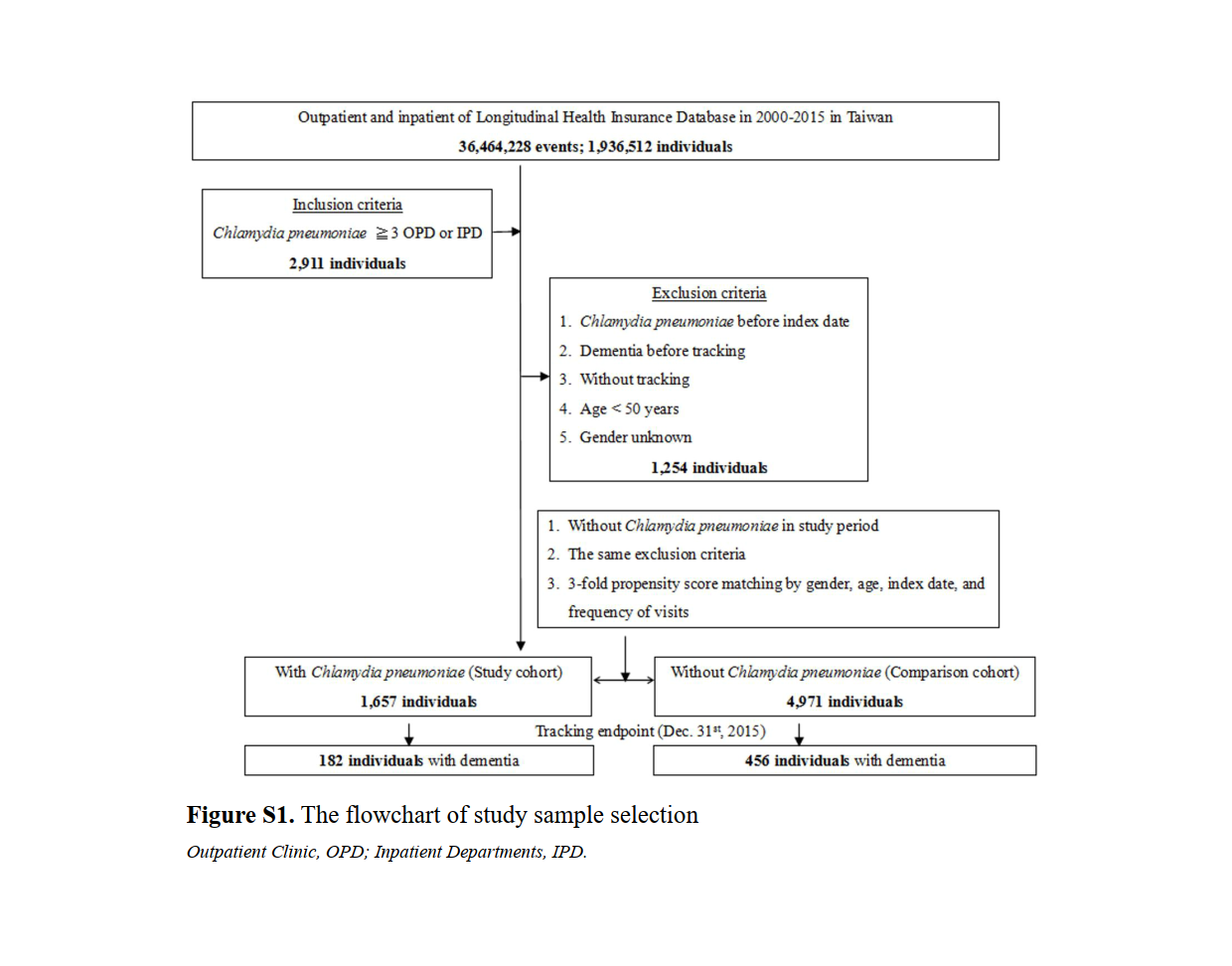

Supplement: Supplementary file 1 [file Image_1.JPEG]
