# Supplementary material for: Association Between Antibiotic Treatment of Chlamydia pneumoniae and Reduced Risk of Alzheimer Dementia: A Nationwide Cohort Study in Taiwan
Source: Front Aging Neurosci. 2021 Aug 18;13:701899. doi: 10.3389/fnagi.2021.701899 (PMC8416516; doi:10.3389/fnagi.2021.701899)
Supplement: Supplementary file 2 [file Table_1.DOCX]

| **Table S1. Factors of dementia stratified by variables listed in the table by using Cox regression and Fine & Gray's competing risk model** | | | | | | | | | | |
| --- | --- | --- | --- | --- | --- | --- | --- | --- | --- | --- |
| ***Chlamydia pneumoniae*** | **With** | | | **Without** | | | **Competing risk in the model** | | | |
| **Stratified** | **Events** | **PYs** | **Rate (per 10^5^ PYs)** | **Events** | **PYs** | **Rate (per 10^5^ PYs)** | **Adjusted HR** | **95% CI** | **95% CI** | ***P*** |
| **Total** | 182 | 19,548.63 | 931.01 | 456 | 59,668.60 | 764.22 | 1.599 | 1.284 | 1.971 | <0.001 |
| **Gender** |  |  |  |  |  |  |  |  |  |  |
| Male | 122 | 12,353.35 | 987.59 | 275 | 34,943.45 | 786.99 | 1.647 | 1.323 | 2.030 | <0.001 |
| Female | 60 | 7,195.28 | 833.88 | 181 | 24,725.15 | 732.05 | 1.495 | 1.201 | 1.843 | <0.001 |
| **Age group (years)** |  |  |  |  |  |  |  |  |  |  |
| 50-64 | 31 | 3,477.99 | 891.32 | 51 | 6,269.98 | 813.40 | 1.438 | 1.155 | 1.773 | <0.001 |
| ≧65 | 151 | 16,070.64 | 939.60 | 405 | 53,398.62 | 758.45 | 1.626 | 1.306 | 2.004 | <0.001 |
| **Marital status** |  |  |  |  |  |  |  |  |  |  |
| Without | 94 | 9,561.48 | 983.11 | 229 | 31,103.90 | 736.24 | 1.753 | 1.407 | 2.160 | <0.001 |
| With | 88 | 9,987.15 | 881.13 | 227 | 28,564.70 | 794.69 | 1.455 | 1.169 | 1.794 | <0.001 |
| **Education (years)** |  |  |  |  |  |  |  |  |  |  |
| <12 | 86 | 11,532.76 | 745.70 | 192 | 30,903.47 | 621.29 | 1.575 | 1.265 | 1.942 | <0.001 |
| ≧12 | 96 | 8,015.87 | 1,197.62 | 264 | 28,765.12 | 917.78 | 1.713 | 1.375 | 2.111 | <0.001 |
| **Insured premium (NT$)** |  |  |  |  |  |  |  |  |  |  |
| <18,000 | 178 | 18,933.28 | 940.14 | 451 | 58,867.20 | 766.13 | 1.611 | 1.293 | 1.985 | <0.001 |
| 18,000-34,999 | 4 | 582.41 | 686.80 | 5 | 752.99 | 664.02 | 1.358 | 1.090 | 1.673 | <0.001 |
| ≧35,000 | 0 | 32.94 | 0.00 | 0 | 48.41 | 0.00 | - | - | - | - |
| **DM** |  |  |  |  |  |  |  |  |  |  |
| Without | 147 | 15,470.66 | 950.19 | 358 | 44,885.50 | 797.58 | 1.564 | 1.256 | 1.927 | <0.001 |
| With | 35 | 4,077.97 | 858.27 | 98 | 14,783.10 | 662.92 | 1.699 | 1.365 | 2.095 | <0.001 |
| **HTN** |  |  |  |  |  |  |  |  |  |  |
| Without | 123 | 13,791.70 | 891.84 | 308 | 40,165.62 | 766.83 | 1.527 | 1.226 | 1.882 | <0.001 |
| With | 59 | 5,756.93 | 1,024.85 | 148 | 19,502.98 | 758.86 | 1.773 | 1.423 | 2.185 | <0.001 |
| **Hyperlipidemia** |  |  |  |  |  |  |  |  |  |  |
| Without | 178 | 18,971.22 | 938.26 | 444 | 57,287.41 | 775.04 | 1.589 | 1.276 | 1.959 | <0.001 |
| With | 4 | 577.41 | 692.75 | 12 | 2,381.19 | 503.95 | 1.804 | 1.449 | 2.224 | <0.001 |
| **Coronary artery disease** |  |  |  |  |  |  |  |  |  |  |
| Without | 162 | 17,209.24 | 941.35 | 416 | 51,487.38 | 807.96 | 1.529 | 1.228 | 1.885 | <0.001 |
| With | 20 | 2,339.39 | 854.92 | 40 | 8,181.22 | 488.92 | 2.295 | 1.843 | 2.829 | <0.001 |
| **Obesity** |  |  |  |  |  |  |  |  |  |  |
| Without | 182 | 19,493.18 | 933.66 | 456 | 59,646.04 | 764.51 | 1.599 | 1.284 | 1.971 | <0.001 |
| With | 0 | 55.45 | 0.00 | 0 | 22.55 | 0.00 | - | - | - | - |
| **Cancer** |  |  |  |  |  |  |  |  |  |  |
| Without | 176 | 17,125.75 | 1,027.69 | 433 | 50,132.18 | 863.72 | 2.246 | 1.804 | 2.769 | <0.001 |
| With | 6 | 2,422.88 | 247.64 | 23 | 9,536.42 | 241.18 | 1.526 | 1.226 | 1.881 | <0.001 |
| **Pneumonia** |  |  |  |  |  |  |  |  |  |  |
| Without | 124 | 12,681.95 | 977.77 | 365 | 44,670.47 | 817.09 | 1.571 | 1.261 | 1.936 | <0.001 |
| With | 58 | 6,866.68 | 844.66 | 91 | 14,998.13 | 606.74 | 1.827 | 1.467 | 2.252 | <0.001 |
| **Depression** |  |  |  |  |  |  |  |  |  |  |
| Without | 175 | 19,320.04 | 905.80 | 449 | 59,360.58 | 756.39 | 1.572 | 1.262 | 1.937 | <0.001 |
| With | 7 | 228.59 | 3,062.21 | 7 | 308.01 | 2,272.62 | 1.769 | 1.420 | 2.180 | <0.001 |
| **Bipolar** |  |  |  |  |  |  |  |  |  |  |
| Without | 181 | 19,510.95 | 927.68 | 455 | 59,533.50 | 764.28 | 1.593 | 1.279 | 1.964 | <0.001 |
| With | 1 | 37.68 | 2,653.75 | 1 | 135.10 | 740.19 | 4.706 | 3.779 | 5.801 | <0.001 |
| **Anxiety** |  |  |  |  |  |  |  |  |  |  |
| Without | 96 | 11,737.59 | 817.89 | 268 | 38,925.34 | 688.50 | 1.559 | 1.252 | 1.922 | <0.001 |
| With | 86 | 7,811.04 | 1,101.01 | 188 | 20,743.26 | 906.32 | 1.594 | 1.280 | 1.965 | <0.001 |
| **Alcohol use disorder** |  |  |  |  |  |  |  |  |  |  |
| Without | 182 | 19,533.89 | 931.71 | 452 | 59,601.91 | 758.36 | 1.613 | 1.295 | 1.988 | <0.001 |
| With | 0 | 14.73 | 0.00 | 4 | 66.68 | 5,998.43 | 0.000 | - | - | 0.976 |
| **Substance use disorder** |  |  |  |  |  |  |  |  |  |  |
| Without | 182 | 19,517.81 | 932.48 | 456 | 59,654.80 | 764.40 | 1.599 | 1.284 | 1.971 | <0.001 |
| With | 0 | 30.82 | 0.00 | 0 | 13.79 | 0.00 | - | - | - | - |
| **Sleep disorder** |  |  |  |  |  |  |  |  |  |  |
| Without | 172 | 19,347.33 | 889.01 | 446 | 59,229.74 | 753.00 | 1.550 | 1.244 | 1.910 | <0.001 |
| With | 10 | 201.30 | 4,967.81 | 10 | 438.86 | 2,278.65 | 2.862 | 2.298 | 3.527 | <0.001 |
| ***Chlamydiae* infection or disease** |  |  |  |  |  |  |  |  |  |  |
| Without | 182 | 19,548.63 | 931.01 | 456 | 59,668.60 | 764.22 | 1.599 | 1.284 | 1.971 | <0.001 |
| With | 0 | 0.00 | - | 0 | 0.00 | - | - | - | - | - |
| **CCI_R** |  |  |  |  |  |  |  |  |  |  |
| 0 | 71 | 9,871.81 | 719.22 | 214 | 34,756.15 | 615.72 | 1.533 | 1.231 | 1.890 | <0.001 |
| 1 | 84 | 6,831.88 | 1,229.53 | 163 | 15,662.87 | 1,040.68 | 1.551 | 1.245 | 1.911 | <0.001 |
| 2 | 18 | 1,989.08 | 904.94 | 49 | 5,752.86 | 851.75 | 1.395 | 1.120 | 1.719 | <0.001 |
| 3 | 7 | 473.78 | 1,477.48 | 26 | 2,252.27 | 1,154.39 | 1.680 | 1.349 | 2.071 | <0.001 |
| ≧4 | 2 | 382.08 | 523.45 | 4 | 1,244.43 | 321.43 | 2.137 | 1.716 | 2.635 | <0.001 |
| **Season** |  |  |  |  |  |  |  |  |  |  |
| Spring | 38 | 4,192.74 | 906.33 | 104 | 13,752.89 | 756.20 | 1.573 | 1.263 | 1.939 | <0.001 |
| Summer | 65 | 5,315.95 | 1,222.74 | 138 | 14,935.28 | 923.99 | 1.737 | 1.395 | 2.141 | <0.001 |
| Autumn | 42 | 5,840.02 | 719.18 | 109 | 16,915.71 | 644.37 | 1.465 | 1.176 | 1.806 | <0.001 |
| Winter | 37 | 4,199.92 | 880.97 | 105 | 14,064.71 | 746.55 | 1.549 | 1.244 | 1.909 | <0.001 |
| **Urbanization level** |  |  |  |  |  |  |  |  |  |  |
| 1 (The highest) | 65 | 5,441.80 | 1,194.46 | 137 | 16,763.84 | 817.24 | 1.918 | 1.540 | 2.365 | <0.001 |
| 2 | 95 | 11,656.35 | 815.01 | 195 | 27,904.56 | 698.81 | 1.531 | 1.229 | 1.887 | <0.001 |
| 3 | 6 | 892.22 | 672.48 | 26 | 3,828.79 | 679.07 | 1.300 | 1.044 | 1.602 | 0.017 |
| 4 (The lowest) | 16 | 1,558.26 | 1,026.79 | 98 | 11,171.41 | 877.24 | 1.536 | 1.234 | 1.894 | <0.001 |
| **Level of care** |  |  |  |  |  |  |  |  |  |  |
| Hospital center | 57 | 6,578.26 | 866.49 | 122 | 19,729.83 | 618.35 | 1.839 | 1.477 | 2.267 | <0.001 |
| Regional hospital | 90 | 10,223.68 | 880.31 | 188 | 27,115.14 | 693.34 | 1.666 | 1.338 | 2.054 | <0.001 |
| Local hospital | 35 | 2,746.69 | 1,274.26 | 146 | 12,823.63 | 1,138.52 | 1.469 | 1.180 | 1.811 | <0.001 |
| **PYs = Person-years; DM: diabetes mellitus, HTN: hypertension, CCI_R: Charlson Comorbidity Index, dementia removed; Adjusted HR = Adjusted Hazard ratio: Adjusted for the variables listed in Table 1.; CI = confidence interval; *P:* Chi-square / Fisher exact test on category variables and t-test on continue variable** | | | | | | | | | | |
